# Direct Thermal Imaging of Domain Wall Hot Spots in LiNbO_3_

**DOI:** 10.1002/smll.202508603

**Published:** 2025-11-20

**Authors:** Lindsey R. Lynch, J. Marty Gregg, Amit Kumar, Kristina M. Holsgrove, Raymond G. P. McQuaid

**Affiliations:** ^1^ Centre for Quantum Materials and Technologies School of Mathematics and Physics Queen's University Belfast University Road Belfast BT7 1NN UK

**Keywords:** domain wall, ferroelectric, joule heating, lithium niobate, piezoresponse force microscopy, scanning thermal microscopy

## Abstract

Ferroelectric domain wall devices offer a promising route to low‐voltage, reconfigurable nanoelectronics by confining currents to nanoscale conducting interfaces within an insulating bulk. However, the potential for resistive heating and unregulated temperature increases due to domain wall conduction remains unexplored. Here, scanning thermal microscopy is employed to directly image hot spots in thin‐film lithium niobate domain wall devices. Piezoresponse force microscopy shows that the hot spots correlate with nanodomain structure, and thermal mapping reveals surface temperature rises of ≈20 K at most, levels that are unlikely to negatively affect device performance. This is due to the moderate electrical conductivity of domain walls, their voltage‐tunable erasure, and distributed current pathways, which inherently limit power dissipation and peak temperatures. Finite element electrothermal modelling indicates that domain walls behave as pseudo‐planar heat sources, distinct from the filament‐based heating typically observed in resistive switching oxides. These findings highlight the potential for domain wall devices as an energy‐efficient, thermally stable platform for emerging memory and logic applications.

## Graphical abstract

Ferroelectric domain walls are conducting interfaces that are fully reconfigurable by electrical signals, leading to conceptually new types of multi‐state memory devices. However, detrimental self‐heating of the devices due to highly confined power dissipation in domain walls can occur. Here, Scanning Thermal Microscopy is used to directly visualize hot spots associated with polar nanodomain structure, with temperatures up to ≈20 K observed.

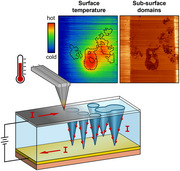

## 1 Introduction

For over a decade, electrically conducting ferroelectric domain walls (DWs) have drawn attention as reconfigurable functional elements that could be used in novel storage memories and neuromorphic device applications. DWs are interfaces that develop naturally at intersections between polar domain variants, and their position can be manipulated through well‐understood voltage‐controlled domain nucleation and growth processes.^[^ ^1^, ^2^ ^]^ DWs are particularly interesting because they can exhibit greater electrical conductivity than the surrounding bulk, facilitated through extrinsic defect engineering of the wall itself, or by introducing a polarization discontinuity that requires compensation by mobile charges.^[^ ^3^, ^4^ ^]^ Although the electrical transport characteristics of conducting walls often appear thermally activated, this remains under some scrutiny,^[^ ^5^ ^]^ with metallic‐like,^[^ ^6^ ^]^ and even superconducting, behaviors having been reported.^[^ ^7^ ^]^ When integrated into demonstrator devices, such as transistors and memristors,^[^ ^8^, ^9^, ^10^, ^11^ ^]^ operation involves using DWs to reversibly make/break contact with electrodes, performing an analogous role to the vacancy filaments that enable resistive switching in transition metal oxides, such as e.g. TiO_2_,^[^ ^12^ ^]^ HfO_2_,^[^ ^13^ ^]^ and TaO_x_.^[^ ^14^ ^]^

Of the ferroelectric systems that are currently known to exhibit enhanced DW electrical conductivity, lithium niobate shows the largest conductance contrast between DWs and bulk.^[^ ^15^ ^]^ This is primarily because the bulk is highly electrically insulating, while the walls have best‐estimate conductivity values in the range of intrinsic semiconductors.^[^ ^15^, ^16^, ^17^ ^]^ Nonetheless, LiNbO_3_ serves as an ideal platform for testing DW device prototypes because it effectively confines device currents to the walls, allowing functionality to be derived entirely from the number density of walls connecting the electrodes. This is not typical, since other ferroelectric systems displaying enhanced DW conductivity can also have non‐negligible bulk conductance, meaning that electrical current pathways are not necessarily well controlled.^[^ ^18^ ^]^ As a result, DW enabled rectification,^[^ ^15^, ^17^ ^]^ transistors,^[^ ^19^, ^20^ ^]^ memristors,^[^ ^21^, ^22^ ^]^ and logic gates,^[^ ^20^, ^23^ ^]^ have been most convincingly demonstrated using LiNbO_3_ integrated into various device geometries. Although most studies have focused on electrical characterization, power dissipation in the current‐carrying DWs will also cause localized Joule heating, which could impair performance or lead to thermal crosstalk in high‐density memory arrays. This is already a known issue for resistive switching in transition metal oxides, where vacancy filament formation and conduction are fundamentally electrothermal processes that depend on self‐heating and local temperature conditions.^[^ ^24^, ^25^ ^]^ However, studying the fundamental properties of the filaments directly is challenging since they can be truly nanoscale, with diameters less than 10 nm. Several factors complicate the direct measurement of filament temperatures via surface thermometry, including lateral heat spreading in the active layer and electrodes, as well as increased sensitivity to interfacial thermal resistances in nanoscale devices.^[^ ^26^, ^27^ ^]^

Over the last few years, the nanoscale spatial resolution of scanning thermal microscopy (SThM) has made it an invaluable tool for direct thermal imaging of filamentary behavior in resistive switching devices. Deshmukh et al.^[^ ^13^ ^]^ were first to investigate local Joule heating of a single filament by SThM in HfO_2_ resistive random access memory devices (RRAMs), revealing surface temperature “hot spots” of over 350 °C, and even higher implied filament temperatures of ≈1300 °C, due to nanoscale confinement of dissipated power. Similar local heating investigations by SThM have subsequently been carried out for NbO_x_ memristors,^[^ ^28^ ^]^ TiO_2_ RRAMs,^[^ ^12^, ^29^ ^]^ and on resistive switching devices based on 2D MoTe_2_.^[^ ^30^ ^]^ As well as revealing the location of filamentary current pathways indirectly through local heating, the studies explore how filament heat generation and spreading can affect performance in realistic device geometries. In this study, we use a combination of SThM and piezoresponse force microscopy (PFM) domain imaging to reveal and quantify the resistive heating associated with ferroelectric DWs under steady‐state current in thin‐film LiNbO_3_ devices. Using SThM, we directly observe hot spot features with surface temperature rises ranging from sub‐Kelvin up to ≈20 K, depending on the dissipated power levels. Unlike in conventional resistive switching systems, we are able to leverage PFM to directly image the distribution of heat sources and correlate this with the observed thermal signals to better understand the relationship between microstructure and device heating.

## 2 Results and Discussion

### 2.1 Device Fabrication and Domain Wall Engineering

**Figure 1:**
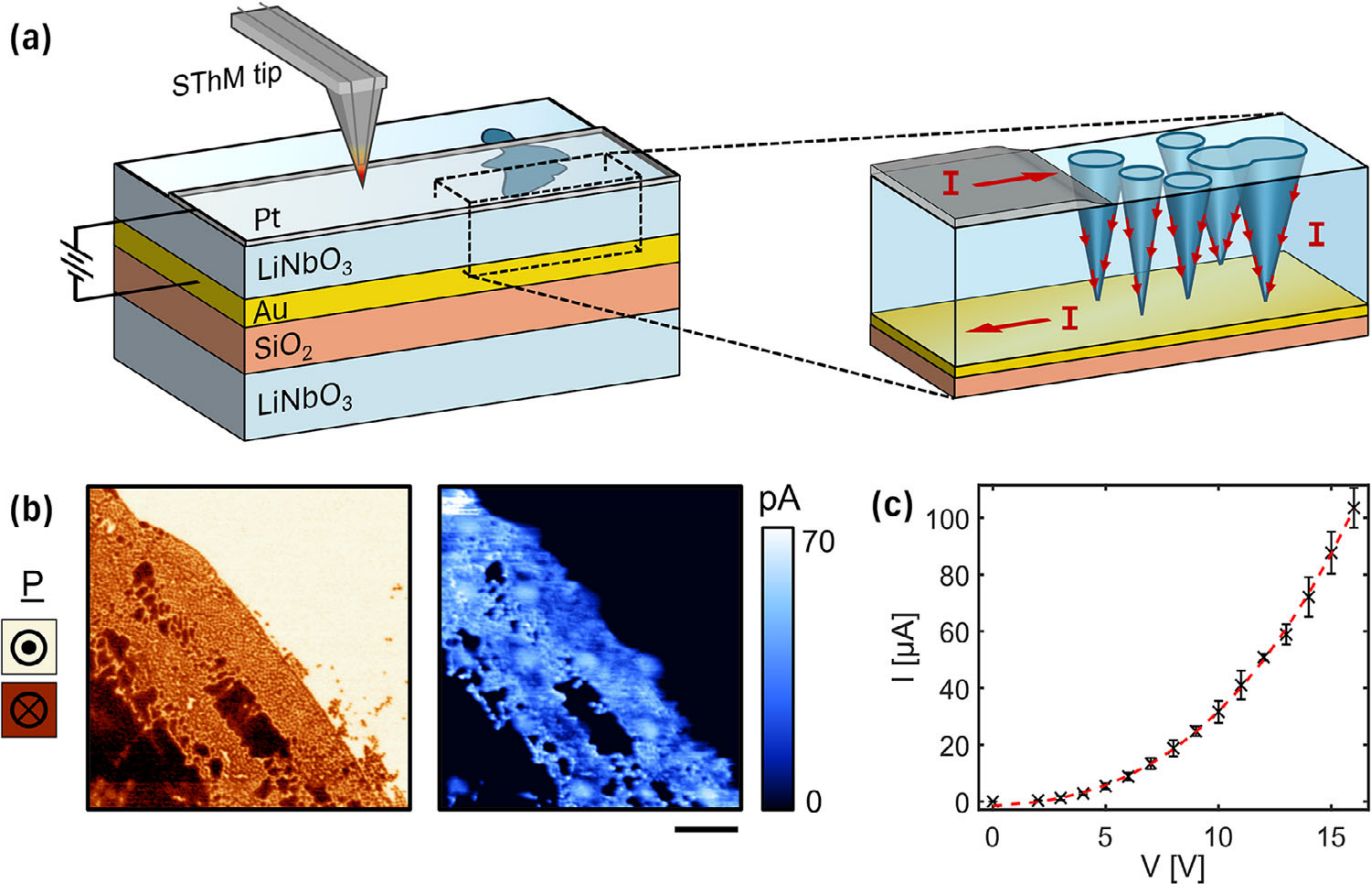
a) Schematic of LiNbO_3_ domain wall device under bias. Scanning thermal microscopy is carried out over the Pt top electrode. Zoom‐in schematically shows the conical domains formed in the top thin‐film LiNbO_3_ layer, with domain walls acting as the only conducting pathways connecting top (Pt) and bottom (Cr‐Au‐Cr) electrodes. b) Typical maps of piezoresponse phase (left) and current (right) that are obtained for polydomain regions on the bare LiNbO_3_ surface. Enhanced conduction is only observed within polydomain regions. Scale bar represents 3 µm. c) Measured *I*–*V* characteristic across the top/bottom electrodes of the domain wall device.

**Figure 2:**
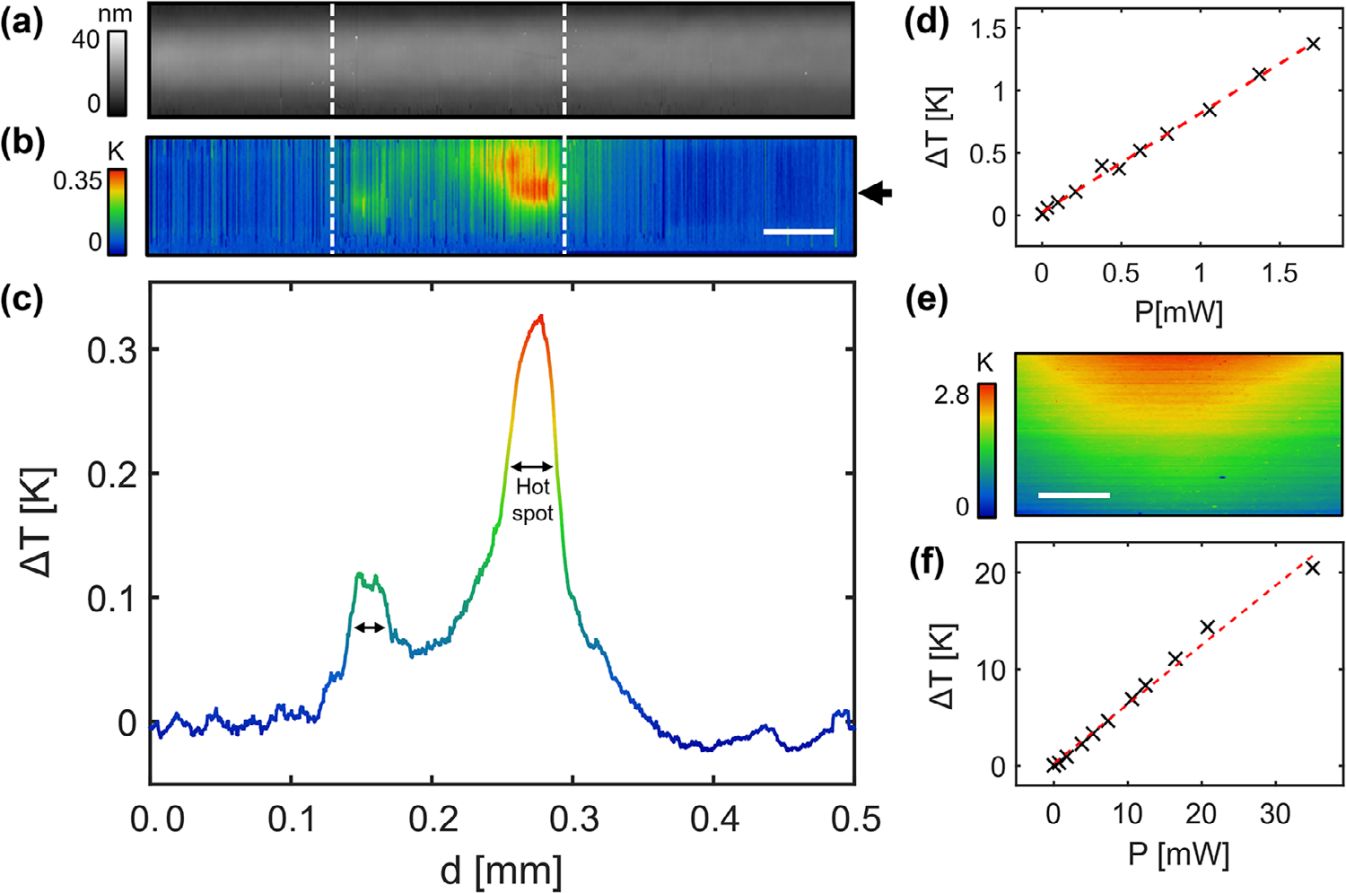
a) 2D topographical map of the Pt electrode deposited on the top surface of the LiNbO_3_ film. The white dashed line demarcates the approximate region on the LiNbO_3_ thin film that was prepared with domain microstructure. b) 2D temperature map obtained using scanning thermal microscopy with device under a constant current of 60 µA. The temperature map was background corrected to remove line noise, highlighting hot spots above the regions of the LiNbO_3_ sample where domain walls were injected. The scale bar measures 50 µm. c) A 1D temperature line profile taken along the long axis of the electrode, indicated by the arrow in (b). The line profile was taken from the background corrected map, (b), and a 4‐point moving average was then applied. The two hotspots are clearly revealed and are indicated by double headed arrows. d) Peak absolute temperature rise of the main hot spot in (b)‐(c), as a function of power dissipated through the device. e) SThM map of a hot spot observed in a different device under a dissipated power of just over 3 mW. The scale bar represents 20 µm. f) Peak absolute temperature vs power for the hot spot in (e).

DW memristor devices (schematized in **Figure** **1**a) were fabricated using commercially available z‐cut thin films of LiNbO_3_ that have been ion‐sliced from congruently grown single crystals and then thermally bonded onto prepared carrier substrates. The structure consisted of 500 nm LiNbO_3_/150 nm Cr‐Au‐Cr/2 µm SiO_2_/0.5 mm LiNbO_3_. In order to engineer the desired microstructure, electrically conducting DWs were injected into the as‐received monodomain LiNbO_3_ thin film layer by an established poling process.^[^ ^21^, ^22^ ^]^ This involved local contacting and biasing of the bare film surface with a removable In‐Ga‐As liquid top electrode. Since the LiNbO_3_ bulk material is highly insulating, it is reasonable to assume that any steady‐state current is due to charge transport along conducting DWs that connect the device electrodes. Therefore, we relied on current feedback to determine the applied bias required to maximize the number of DWs injected under the liquid electrode. An example of the typical domain microstructure obtained by this process is revealed in PFM and conductive‐atomic force microscopy (c‐AFM) mapping of the bare surface, shown in Figure 1b. Regions of densely packed nanodomains are revealed, and spatially resolved current mapping (Figure 1b) shows enhanced electrical conductivity within the polydomain region. This is consistent with previous reports in lithium niobate,^[^ ^21^, ^22^, ^31^, ^32^ ^]^ where enhanced conductivity is attributed to mobile charge transport along charged DWs. More broadly, enhanced electrical conductivity along DWs in ferroelectrics is a well‐established phenomenon that can be explained by a combination of defect‐aggregation‐enabled transport, bandgap reduction at walls, or compensatory charge accumulation at electrostatically charged interfaces.^[^ ^4^ ^]^ The latter mechanism is key for z‐cut lithium niobate thin films, where intermediate switched states involve high spatial densities of charged needle‐like domains, which have previously been described as being like truncated cones due to the large angle of inclination (≈10°) of the DWs.^[^ ^16^ ^]^ This domain morphology is consistent with that expected for the transition from the forward growth phase^[^ ^2^ ^]^ of switching to the early stages of sideways growth. Importantly, since our c‐AFM mapping indicates that current can be passed through DWs, this means that the tips of the domain needles have reached the opposite electrode and formed continuous conducting pathways.^[^ ^22^ ^]^ It is important to note that it is solely the DWs (interfaces) that carry current, and not the domain volume. The observed smearing of current signal in Figure 1b is due to limitations in imaging resolution, and further current mapping is shown in Figure S1 (Supporting Information) to emphasize that the signals originate from the walls. For the device, a long platinum top electrode of 35 nm thickness and lateral dimensions of 62.5 µm by 1 mm was deposited on top of the prepared domain structure for device measurements and thermal investigation. The domains spanned a 160 µm long region under the electrode, demarcated by the white lines in **Figure** **2**a. Therefore, when the top electrode is biased at one end, charge flow is confined to within the bar (since the monodomain film contacted underneath is highly insulating) until the DWs are reached, which provide leakage paths to the grounded bottom electrode (see Figure 1a inset). Since the DWs in thin‐film LiNbO_3_ are considered to be semiconducting,^[^ ^16^, ^17^ ^]^ they should present the highest electrical resistance contribution in the circuit. Therefore, there is an expectation for localized Joule heating within the DWs that pass current between the top and bottom electrodes. Forward‐bias current‐voltage measurements of the prepared DW device are shown in Figure 1c, with steady‐state currents measured up to 100 µA. These currents are maintained at a low level to maintain device integrity and to avoid electric field‐induced domain switching.

### 2.2 Initial Observations of Local Heating in LiNbO_3_ Domain Wall Device

To obtain an initial overview of the device self‐heating, SThM was carried out over the length of the top electrode while under a current of 60 µA (0.66 mW power), with the topography map of the region shown in Figure 2a. The raw temperature data were background corrected (see Figure S2 and Note S1, Supporting Information) to minimize sporadic noise that arises from changing tip‐surface conditions during the scan, resulting in the temperature profile shown in Figure 2b. A region of elevated temperature is clearly observed, which lies within the central area that was prepared with domain microstructure (demarcated by the white dashed lines). This would strongly suggest that current leakage through the DWs, and associated Joule heating, is responsible for the detected hotspots. The decay in temperature away from the center to either end of the electrode further implies that the buried DWs are the main source of heat in the device. Taking a line section of surface temperature along the center of the electrode long axis (Figure 2c) details a primary “hot spot” feature with a modest local relative temperature rise of ≈0.3 K. A neighboring hot spot can also be identified, which is smaller in area and has a smaller temperature rise of less than 0.1 K. The peak temperature of the larger hot spot scales linearly with the dissipated power (determined using two‐probe current and voltage readings), adding confidence that the observed signals have their origin in Joule heating (see Figure 2d). To identify if self‐heating of the top electrode is a significant contributor to the observed temperature increases, we also measured Joule heating for currents directed solely through the top electrode (see Figure S3, Supporting Information), finding this to be a negligible contribution compared to resistive heating by the DWs. We have also observed larger hot spot peak temperatures of just over 20 K for proportionately larger dissipated power (≈35 mW) in another similarly prepared DW device, shown in Figure 2e,f, and further discussed in Figure S4 (Supporting Information).

### 2.3 Correlation Between Domain Structure and Hot Spot Formation

**Figure 3:**
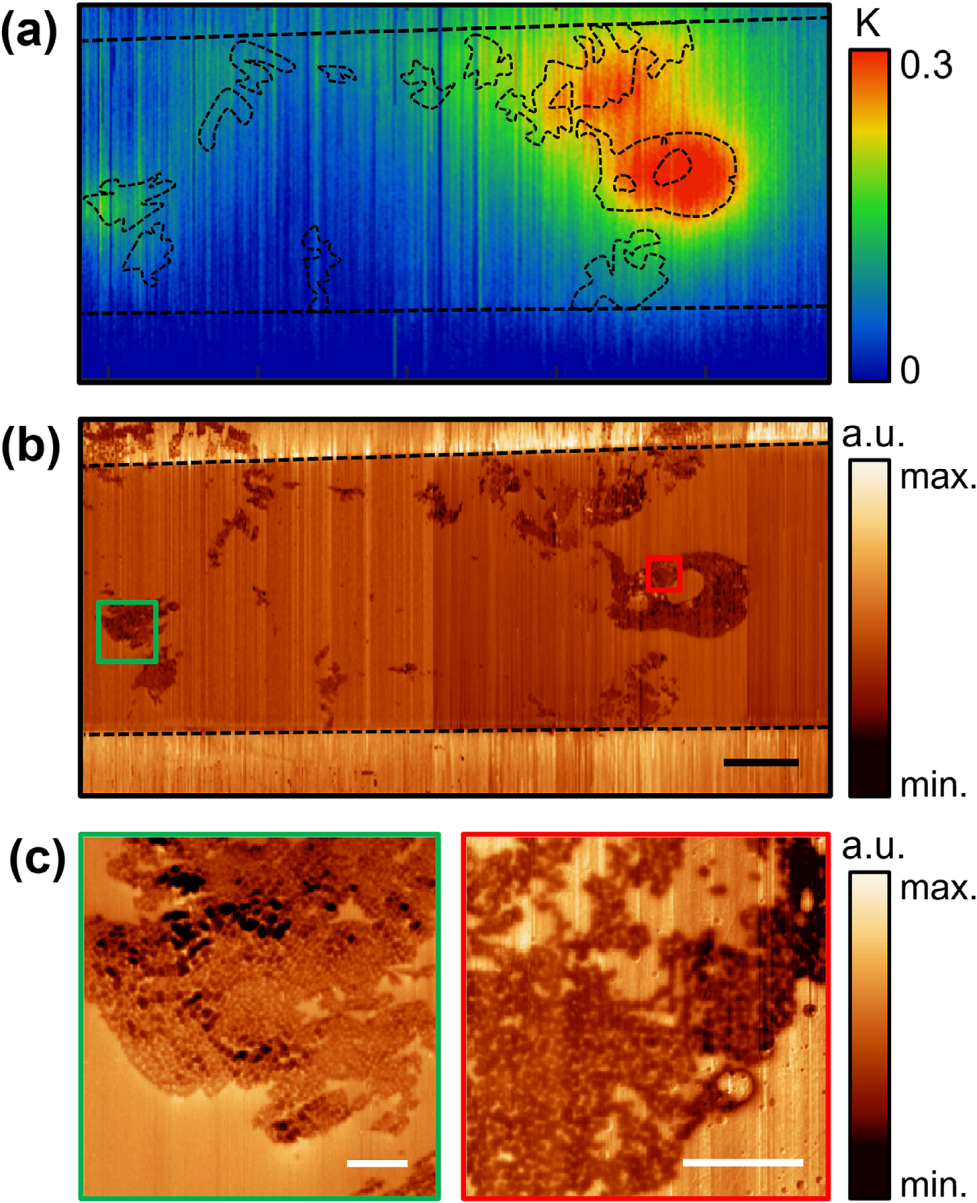
a) The hotspot region mapped using scanning thermal microscopy, overlaid with the perimeter of the partially switched domain regions extracted from through‐electrode piezoresponse force microscopy data shown in b). The horizontally running dashed black lines indicate the edges of the top electrode. The locations of the hotspots correspond to polydomain regions in the LiNbO_3_, indicating that the elevated temperatures are due to domain wall Joule heating. The scale bar represents 20 µm. c) Higher resolution scans corresponding to the green and red boxed regions in (b). The polydomain regions contain a high density of closely packed nanodomains. The scale bars represent 2 µm.

To investigate the relationship between the spatial distribution of DWs and observed heating signals, we carried out polar domain mapping surrounding the hot spot location for the device shown in Figure 2a,b. Higher resolution thermal mapping of the hot spots is shown in **Figure** **3**a, with through‐electrode PFM of the same region in Figure 3b. We extracted contours of the perimeter of written domains from the PFM, and superimposed these on the temperature map, showing that the temperature peaks previously identified in Figure 2b,c can be associated with underlying domain microstructure in the ferroelectric thin film. Higher resolution imaging of the polar microstructure in these regions (Figure 3c) reveals a densely packed arrangement of nanodomains, which were not immediately apparent from the lower resolution survey scan in Figure 3b because of their small size, with diameters of 100–150 nm. The nanodomain morphology suggests that the walls have a through‐depth conical aspect, as indicated by cross‐sectional electron microscopy in previous investigations of conducting DWs in these commercial thin films.^[^ ^16^, ^22^ ^]^

**Figure 4:**
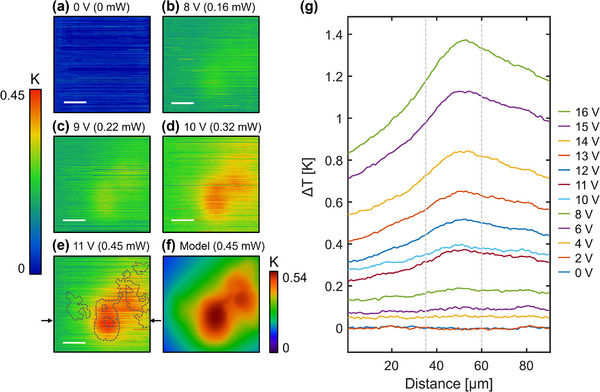
a–e) Spatial maps of temperature change obtained as a function of power dissipated, showing the development of hotspots. Outline of domain wall region obtained from piezoresponse force microscopy data is overlaid on (e). Scale bars represent 20 µm. f) Surface temperature map generated by finite element heat flow modelling. g) Line sections of temperature for a slow dc voltage sweep taken along the arrowed location indicated in (e). The vertically running dashed lines indicate the edges of the polydomain region. A moving average has also been applied to the data in (g).

In **Figure** **4**, we examine the spatial development of the main DW hot spot with increasing device power. The temperature map for the device in the power off state is shown in Figure 4a, and local heating is mapped for increasing dissipated power on the order of 0.1 mW in Figure 4b–e. Initially, as the bias is increased above 0 V, local heating begins to occur and the hot spot becomes only faintly observable above background (by ≈60 mK in Figure 4b), likely due to the prevalence of heat spreading into the surrounding material. It is worth noting that the heating observed at these lower voltages still originates from power dissipated in the DWs and not due to self‐heating of the top electrode (see Figure S3, Supporting Information). As power is increased further (Figure 4d,e), the localized heating becomes more obvious and the temperature maps begin to display hot spot features that resemble the perimeter of domains seen in the PFM map (overlaid in Figure 4e). This behavior is also captured in Figure 4g temperature line sections, which show the hot spot emerging above the background during a slow D.C. voltage sweep, where enough time was given for the tip‐sample system to thermally equilibrate at each step. Comparing the PFM and SThM maps, we see that there is not a one‐to‐one match between the domain microstructure and the heating signals, even at the highest power. A lack of hot spots in regions where domain coverage is sparse may be suggestive of a critical DW density and threshold for local power dissipation to generate detectable hot spots. Alternatively, these DWs may not actually be carrying electrical current, due to poorly formed domain structures that do not connect the electrodes or inherent complexity in the current percolation pathway.^[^ ^33^ ^]^ Changes in the hot spot morphology with increasing voltage are primarily due to increases in the locally dissipated power, not by bias‐induced changes in the microstructure (i.e., nucleation and growth of polar domains). We verify this by PFM domain imaging carried out before and after the thermal mapping, which shows no noticeable changes in the microstructure (Figure S5, Supporting Information). Nonetheless, it is important to note that polarization switching can be triggered by sufficiently large voltages (>25 V) and will place an upper limit on the achievable power density and associated self‐heating that can be achieved. This is facilitated by domain growth and coalescence, which reduces the DW number density, and results in a progressively increasing device resistance until switching is complete and no current leakage pathways remain.

### 2.4 Electrothermal Modelling and Implications for Device Performance

To rationalize the steady‐state temperature patterns observed experimentally by SThM, we have carried out finite element electrothermal modelling where the PFM domain maps were used as input to recreate the DW distribution (for details, see Experimental Section). We modelled the DWs as 2D current‐carrying interfaces and used DW electrical conductivity as a fitting parameter to match the experimentally measured device resistance. The modelled surface temperature map for the biasing conditions in Figure 4e is shown in Figure 4f, and further finite element modelling is detailed in Figure S6 (Supporting Information). Even though the heat originates from a nanostructured arrangement of DWs, the resulting modelled hotspot temperature distribution varies smoothly on the microscale, and the main features are captured in our SThM maps. The good qualitative match between model and experiment, therefore, indicates that the DWs can reasonably be considered to behave as planar heat sources buried within the surrounding inactive bulk, which is conceptually distinct from filament‐based models used to describe resistive switching in binary oxides. For the device geometry studied, there is no significant difference between the interior temperature of the DWs and the top electrode surface temperature, even when reasonable values for interfacial thermal resistances between the electrodes and LiNbO_3_ are assumed (see Figure S7 and Note S2, Supporting Information). To explore the effect of downscaling on the running temperature, a 1 × 1 µm device is modelled in Figure S8 (Supporting Information), which results in an average current density of ≈118 nA µm^2^ for 5 V operation (comparable in performance to Pb(Zr,Ti)O_3_ DW devices).^[^ ^34^ ^]^ In this case, the dissipated power of 0.59 µW is associated with only a small peak temperature rise of ≈20 mK. From a practical point of view, it therefore seems unlikely that the large temperature increases seen in filamentary systems (potentially up to 10^3^ K)^[^ ^13^, ^25^ ^]^ would arise in DW memristors, unless devices can be configured for high‐power operation through further enhancements in DW electrical conductivity (Figure S6d, Supporting Information), or optimized device designs.^[^ ^35^ ^]^ This is necessary because deletion of DWs at super‐coercive biases typically places an effective upper limit on the current that can be forced through the device. In addition, the web‐like geometry of DWs helps to distribute current and avoid thermal bottlenecks, as can occur in isolated filaments due to extreme power confinement. The expectation for low running temperatures has positive implications for reliability, especially since LiNbO_3_ DW currents can become unstable and decay in time if the temperature is elevated above 70 °C.^[^ ^15^ ^]^ From a power efficiency standpoint, the use of LiNbO_3_ is advantageous because device electrical conductance can be tuned by minor adjustment of wall inclination angle using sub‐coercive reverse bias, therefore enabling reproducible resistance control without resorting to energy‐inefficient polarization cycling.^[^ ^21^ ^]^

## 3 Conclusion

In conclusion, we have used SThM to spatially map the local heating that occurs in LiNbO_3_ DW memristor devices under steady‐state current. We find clear evidence of local hot spots developing on the electrode surface, with peak temperature rises ranging from sub‐Kelvin to 20 K, proportionate to the magnitude of device current. By using PFM to image the polar microstructure directly, we verify that these heating signals are due to power dissipated within the conducting DWs. However, not all observed nanodomain regions are associated with hot spots, due to either insufficient local heating or because some DWs do not contribute to electrical current leakage. Electrothermal finite element modelling shows that the mapped surface temperature distribution can be broadly reproduced by treating the DWs as pseudo‐planar heat sources, which is qualitatively distinct from that of filamentary systems. DW devices offer unique advantages through structural tunability and potential for low‐voltage operation, while their comparatively low thermal footprint minimizes the risk of thermal degradation and crosstalk. Furthermore, combined SThM and PFM investigations (also demonstrated in^[^ ^36^ ^]^) can provide direct thermal‐structural correlations that are not easily accessed in conventional filament‐based systems. Finally, deploying DWs as configurable planar heaters may open up opportunities for customizable microscale heating,^[^ ^37^ ^]^ with potential applications in nanomaterials synthesis,^[^ ^38^ ^]^ particle manipulation,^[^ ^39^, ^40^ ^]^ and electrothermal actuation.^[^ ^41^ ^]^ In such contexts, spatially selective heating via voltage‐controlled DW patterning could offer a lithography‐free alternative for fabricating complex heater arrays.^[^ ^42^ ^]^

## 4 Experimental Section

### Piezoresponse Force Microscopy and Current‐AFM Mapping

All scanning probe microscopy measurements were carried out using an Oxford Instruments MFP‐3D Asylum atomic force microscope. For PFM and c‐AFM, Pt/Ir‐coated Si probes (Nanosensors model PPP‐EFM tips) were used, with a 2 V_ac_ potential difference applied to the probes at 330–340 kHz for PFM and 10 V_dc_ for c‐AFM.

### Scanning Thermal Microscopy

Spatially resolved temperature mapping of the self‐heated device was carried out using SThM mode on an Oxford Instruments MFP‐3D Asylum atomic force microscope. Kelvin Nanotechnology model KNT‐SThM‐2an probes were used, which comprise of a silicon nitride cantilever with a palladium track micropatterned onto the tip. As the tip was rastered over the surface, maps of tip resistance are obtained using an in‐built Wheatstone bridge sensing circuit, which can be converted into maps of surface temperature using a calibration factor determined from SThM measurements made on an independently temperature‐controlled Pt surface. Further details on the tip calibration are presented in Figure S9 and Note S4 (Supporting Information).

### Electrothermal Finite Element Modelling

Finite element modelling was carried out for a 3D geometry using the Electric Currents and Heat Transfer interfaces in COMSOL Multiphysics software package. The modelled thin film layer thicknesses matched those of the sample used in the experiment, and the Pt bar dimensions were chosen as 62.5 × 193 × 0.04 µm^3^. The modelled substrate dimensions of 260 × 140 × 50 µm^3^ were chosen as a tradeoff between recreating the real bulk geometry and considerations of computational expediency. All external geometry faces were assigned adiabatic heat flow conditions, except for the substrate base, which had a fixed temperature condition. COMSOL material files were used as input for the electrical and thermal properties of LiNbO_3_, SiO_2,_ and the buried Cr‐Au‐Cr electrode was treated as elemental Au. The electrical and thermal conductivities of the Pt thin‐film top electrode were reduced from elemental values by a factor of 10, based on two‐probe resistance measurements and assuming adherence to the Weidemann–Franz law. The DWs were modelled as internal interfaces assigned with the “electrical shielding” boundary condition, enabling 2D electrical currents between the top and bottom biased electrodes. The electrical conductivity of the DW interfaces was used as a fitting parameter so that the modelled current and voltage values (and hence power) matched the experimentally measured ones. This was achieved by assuming a DW conductivity of σ_0_ = 0.35 S m^−1^. This value for σ_0_ should not be overinterpreted, as it depends on assumptions about DW density and subsumes the effect of any electrical contact resistances between the electrode layers and LiNbO_3_ thin film. The COMSOL heat transfer interface was used for generating 3D temperature maps associated with the Joule heating, from which surface temperature profiles on the Pt electrode and within the LiNbO_3_ layer were extracted. The recreation of the hot spot morphology observed by SThM is encouraging (Figure S6, Supporting Information), considering the range of assumptions made in the model, including: the thermal properties of each layer, the spatial density and morphology of DWs, the DW electrical conductivity, no electrical interfacial resistances accounted for, or any possible confounding role played by the SThM tip.

## Conflict of Interest

The authors declare no conflict of interest.

## Author Contributions

L.R.L. performed all of the sample preparation, scanning probe microscopy experiments and finite element modelling. The study was conceived of by R.G.P.M. R.G.P.M. and A.K. realized the SThM capability needed for the experiments. R.G.P.M. and K.M.H. supervised the research. The manuscript was written mainly by R.G.P.M. and L.R.L., who were also primarily responsible for data analysis and interpretation. All authors contributed to the discussion and interpretation of results, and were involved in the manuscript editing.

## Supporting information



Supporting Information

## Data Availability

The data that support the findings of this study are openly available at the following URL/DOI: https://doi.org/10.17034/e314054a‐1735‐405f‐9db6‐d1a9b2131150
