# Supplementary material for: Direct Thermal Imaging of Domain Wall Hot Spots in LiNbO3
Source: Small. 2025 Nov 20;22(1):e08603. doi: 10.1002/smll.202508603 (PMC12757977; doi:10.1002/smll.202508603)
Supplement: Supplementary file 1 — Supporting Information [file SMLL-22-e08603-s001.pdf]

NANO · MICRO small

Supporting Information

for *Small*, DOI 10.1002/smll.202508603

Direct Thermal Imaging of Domain Wall Hot Spots in $LiNbO_3$

Lindsey R. Lynch, J. Marty Gregg, Amit Kumar, Kristina M. Holsgrove and Raymond G. P. McQuaid**

Supporting Information

Direct Thermal Imaging of Domain Wall Hot Spots in $LiNbO_3$

Lindsey R. Lynch, J. Marty Gregg, Amit Kumar, Kristina M. Holsgrove and Raymond G. P. McQuaid**

Centre for Quantum Materials and Technologies, School of Mathematics and Physics, Queen's University Belfast, University Road, Belfast BT7 1NN, United Kingdom

E-mail: llynch14@qub.ac.uk, r.mcquaid@qub.ac.uk

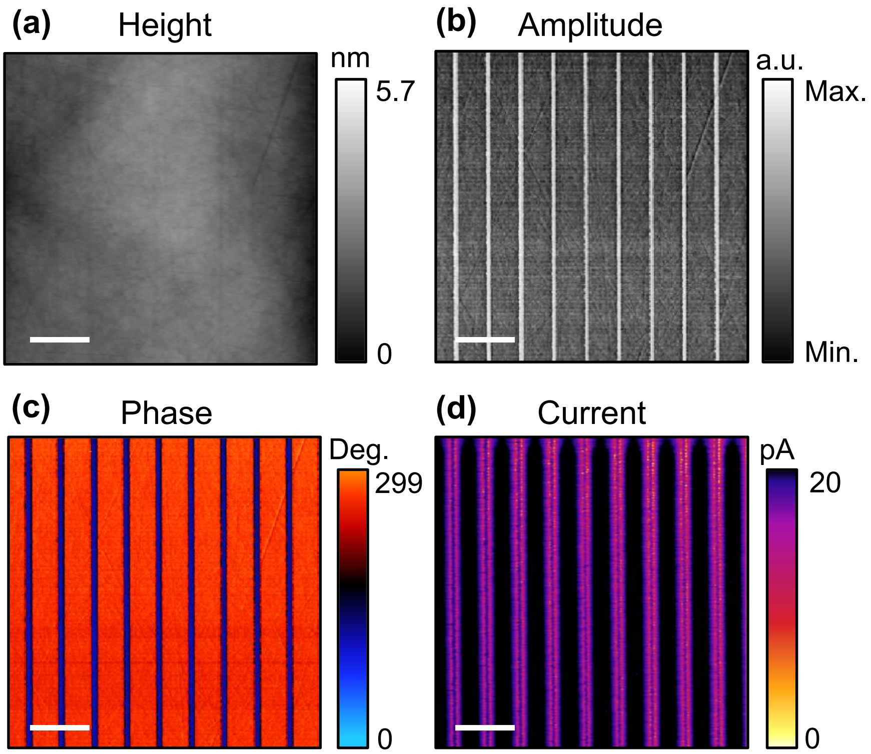

Figure S1. (a) Topography map of bare $LiNbO_3$ thin-film surface. (b) Vertical-mode piezoresponse force microscopy amplitude and (c) phase for an engineered domain structure. (d) Current map obtained using the conductive-atomic force microscopy mode, showing that current signals originate from the domain walls. Scale bars represent 3 µm.

Note 1: Background correction of SThM temperature maps

In the main manuscript, the temperature data presented in Figure 2b,c and Figure 4a-e have been background corrected to reveal the hot spot morphology. In the raw scanning thermal microscopy (SThM) temperature map, shown in Figure S2a, there are noticeable line-by-line

jumps in signal that are highly unlikely to be associated with real temperature changes. These jumps can be seen in the Figure S2b line profile '(1)', where a smooth temperature decay might ordinarily be expected as distance is increased from the buried heat source (the hot spot in the center). Changing tip-sample contact conditions from one scan line to the next (a common issue in scanning probe microscopy methods) is the most likely source of these sporadic signals. However, any changes in tip-sample contact are unknown and challenging to quantify without significant added complexity to the measurement.[1,2] Therefore, our approach is to take the horizontally-running temperature line section along the long edge of the SThM montage as an approximated background correction (indicated by arrow '(2)' in Figure S1a). The idea here is that, if there are any erroneous offsets included in the central line section of temperature (line '(1)' in Fig S2b), then the same erroneous offsets will be captured in the line section taken along the edge of the scan montage (line '(2)' in Fig S2b). This is based on the assumption that the erroneous signal offset remains the same along any given vertically-running SThM line in Figure S2a. Subtracting the temperature line profile '(2)' from '(1)' gives the corrected temperature profile in Figure S2d, which reveals a more smoothly decaying profile away from the location of the hot spot peak temperature (a four-point moving average has been applied also). Further to this, the same background trace can be removed from all line sections of temperature along the bar's long axis, generating the corrected 2D temperature map shown in Figure S2c, where the morphology of the hot spot is more clearly revealed. However, the correction is not perfect: as well as removing erroneous offset signals, any signals associated with real temperature rises along the edge of the bar are also removed. Looking at the background trend in Figure S2b, there is also a slowly varying temperature background rise of ~ 0.2 - 0.4 K, which is removed by the correction alongside the rapidly varying noise signals, likely leading to an underestimation of the hot spot peak temperature in corrected maps. Nonetheless, this approach is a satisfactory compromise for revealing the morphology of the hot spot and the associated temperature rise relative to the immediately surrounding area.

For Figure 4a-e in the main manuscript, the same background correction approach is applied to remove sporadic line-by-line noise. However, in this case the background temperature is quite uniform so an average temperature value is added to each corrected map. This means the SThM maps in Figure 4a-e can be considered as *absolute* temperature rise rather than *relative* temperature rise, as in the case of Figure 2b and c. We also note that four-point moving averaging has been applied to Figure 2c and Figure 4g of the main manuscript but has not been applied to any other line sections in the manuscript.

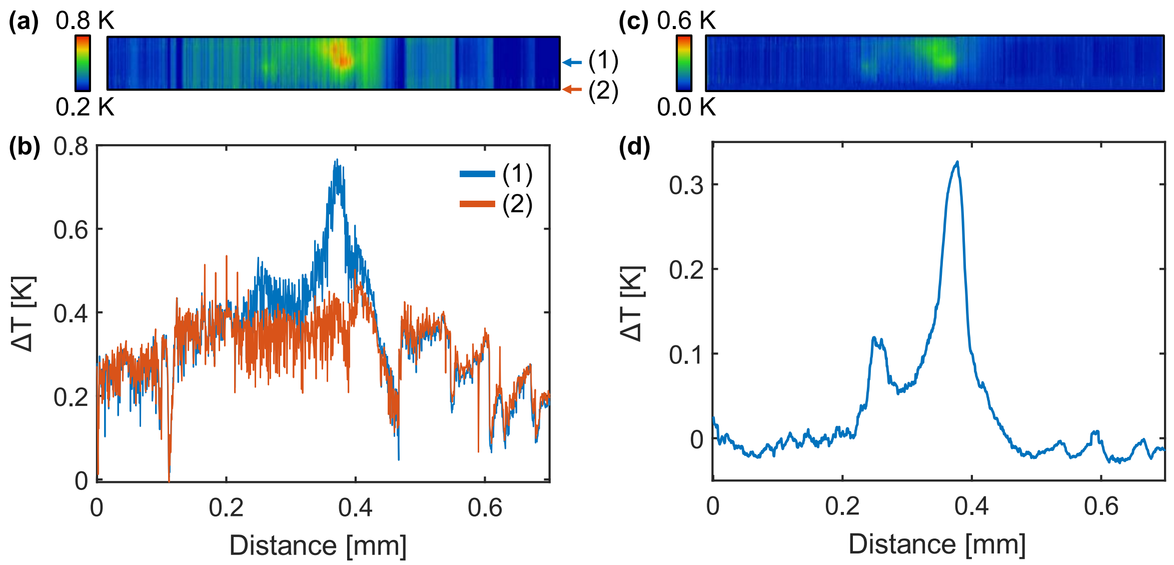

Figure S2. (a) As-obtained temperature map on the Pt top electrode measured by scanning thermal microscopy. (b) The blue line '(1)' is a 1D line section of temperature taken along the central axis of the 2D surface temperature map shown in (a), indicated by arrow '(1)'. The orange line '(2)' is taken along the bottom edge of the same 2D temperature map, indicated by arrow '(2)' in (a). The orange line profile is treated as a background signal and removed from each line along the long axis of the 2D map, giving the corrected temperature map in (c). (d) The corrected temperature line section along the central axis which has also been smoothed by a four-point moving average.

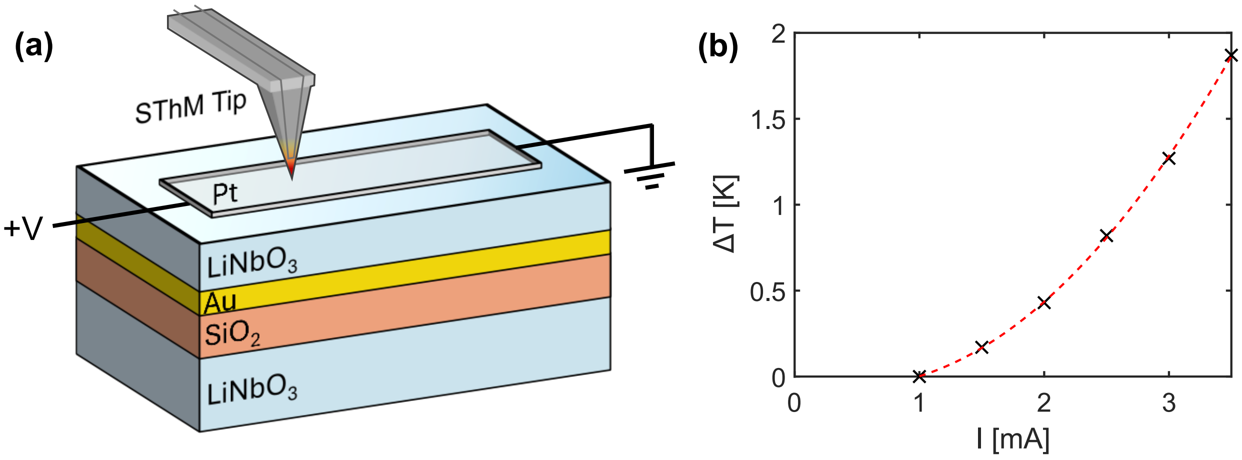

Figure S3. (a) Schematic of electrode bar deposited on top of a monodomain $LiNbO_3$ thin-film. Note that the bar is voltage signalled at either end, ensuring current is confined to within the cross section of the bar. (b) The resulting temperature rises measured on the surface of the electrode as a function of current. Note that the maximum current measured in the main manuscript is significantly less than 1 mA and therefore that the contribution from self-heating within the electrode itself is considered to be negligible.

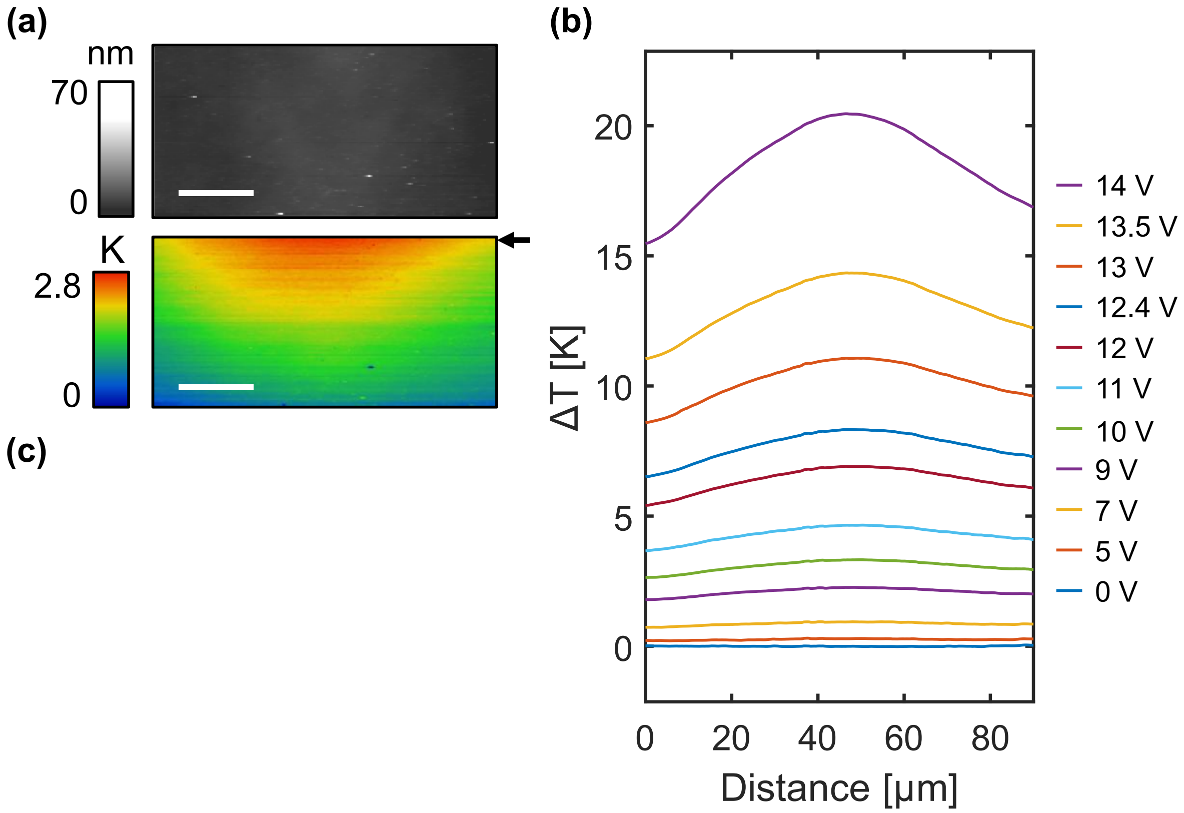

Figure S4. (a) Topography (top panel) and temperature map (bottom panel) of a hotspot region on the top electrode in a different domain wall device, under 9 V bias (3 mW power). Scale bars represent 20 μm. (b) Temperature line sections of the hot spot, taken along the arrowed line in (a), examined under increasing bias. (c) Peak hotspot temperature as a function of power dissipated in the device. Temperature rises of 20.5 K are measured on the surface for a current of 2.5 mA, which is driven by a sub-coercive voltage of 14 V. No background corrections have been applied to any data in this figure, all ΔT values are absolute temperature rises above ambient.

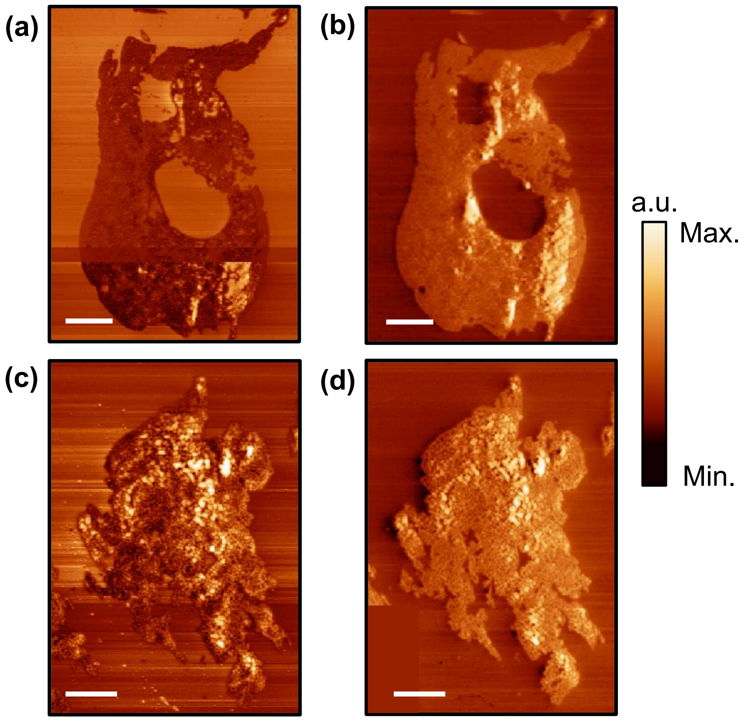

Figure S5. Through-electrode piezoresponse force microscopy imaging of the switched domain region responsible for the main hotspot before, (a), and after, (b), extensive thermal investigations. During thermal experiments, the device was subject to varying levels of sub-coercive forward bias on timescales from minutes to hours. Despite changes in the apparent amplitude contrast across domain variants (a common imaging artefact arising from the optical beam deflection measurement of the cantilever)[3], the form of the domain microstructure appears to be unchanged. Scale bars represent 5 µm. Panels (c) and (d) show another region before and after experiment, which similarly remains unchanged. The development of hot spot morphology is therefore primarily driven by increases in dissipated power rather than by changes in the domain microstructure and the associated current leakage pathways. Scale bars represent 3 µm.

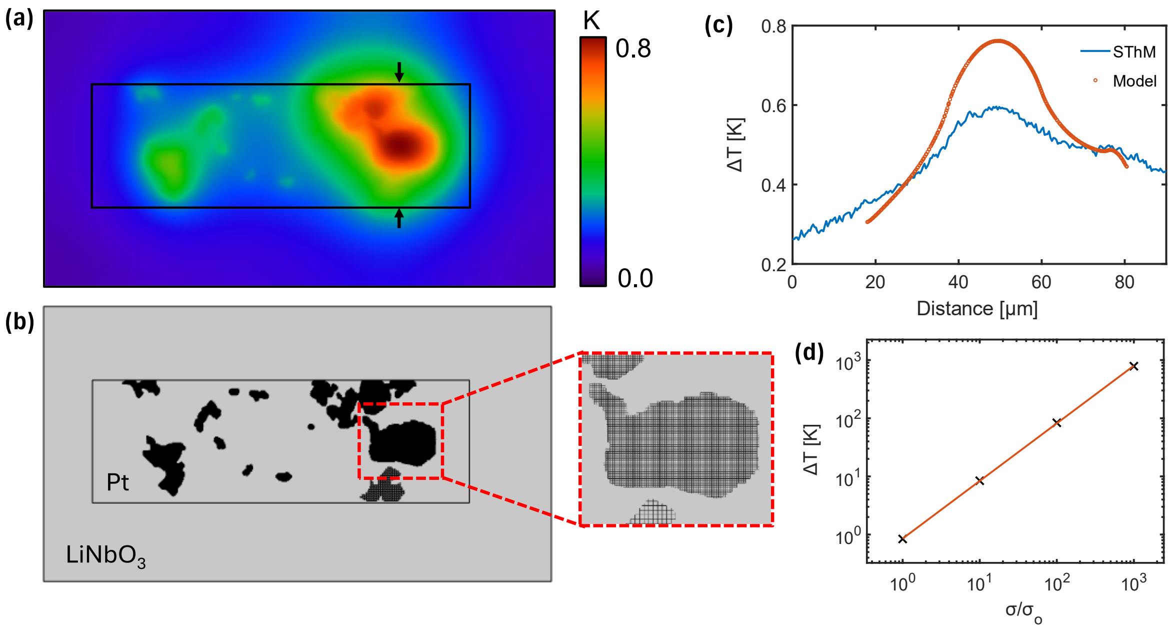

Figure S6. (a) Modelled temperature map on the top surface of the sample for the model geometry shown in (b). Inset details square grid arrangement of 2D interfaces (within the $LiNbO_3$ layer) with spacings of 500 nm (central region) and 1 μm (lower region) to approximate the domain wall configuration. (c) Comparison of temperature line section through the hot spot (taken on the surface electrode along the arrowed line section in (a)) with the experimental data, for similar power dissipated. (d) Modelling higher domain wall conductivities results in proportional increases in temperature (due to increased current drawn for equivalent bias).

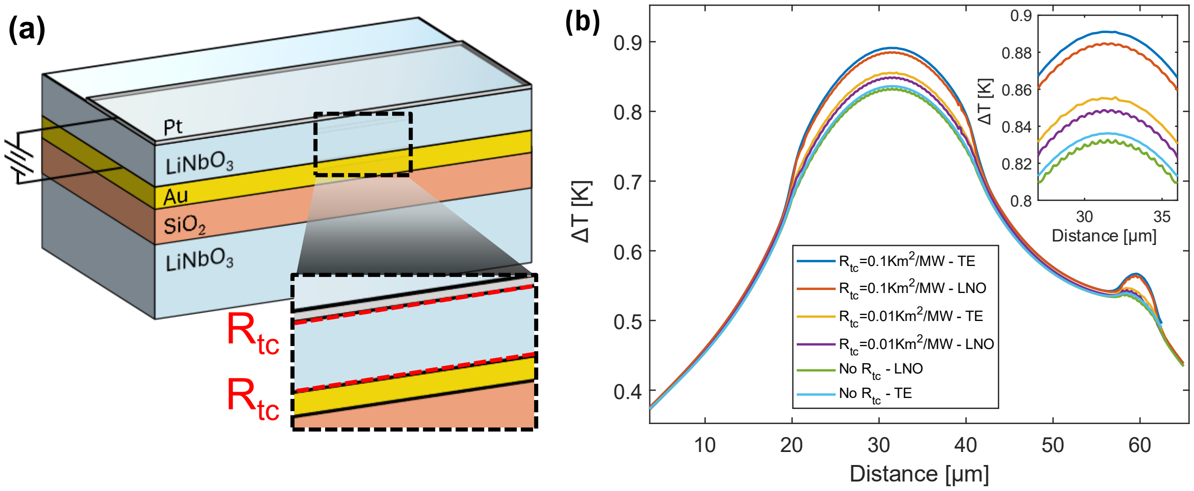

Figure S7. (a) Schematic of sample layer geometry with inset indicating where interfacial contact resistances (R_{tc}) are included in the model. (b) Line section of modelled temperature taken along the central axis of the wire, intersecting the main hot spot (at distance 30 μm) and a smaller one (at distance 60 μm). The pairs of line sections are taken on the top surface of the electrode (legend TE) and through the center of the $LiNbO_3$ film (legend LNO), so that the discrepancy between temperatures in the respective layers, due to R_{tc}, can be examined.

Note 2: Effect of Thermal Boundary Resistance on Domain Wall Temperature

In nanoscale devices, the effect of reduced dimensions on heat dissipation can mean that the effective thermal resistance of the device becomes dominated by interfacial contributions, leading to significantly increased local temperatures.[4] This is important for resistive switching nanodevices, where interfacial resistances can cause the temperature of buried current-carrying filaments to be increased by several factors compared to the measured surface temperatures.[5] To assess the possible role of thermal boundary resistances (R_{tc}) on domain wall temperatures, we repeated the model from Figure S6 and introduced non-zero R_{tc} values for the top electrode (TE)-$LiNbO_3$ interface and for the $LiNbO_3$-bottom electrode (BE) interface. These values are *a priori* unknown, so we explored a range of values of R_{tc} between 0.01 - 0.001 Km^2MW^{-1}, which captures the range of typically encountered values in real thin-film interfaces.[4] The purpose of the simulations is to identify whether the true temperature of the buried domain walls could be significantly larger than the surface temperature measured by SThM. From Figure S7, it can be seen that the influence of the thermal boundary resistances is relatively minor, leading to negligible differences between surface temperature and $LiNbO_3$ layer temperature. In these models, the main effect of the R_{tc} is to increase the absolute temperature rise in the $LiNbO_3$ layer (and concurrent surface temperature) by only

~10%. Therefore, the surface temperatures are likely to be a good representation of the interior temperature in the $LiNbO_3$ layer with the dominant heat loss being predominantly through the surrounding film and the supporting substrate.

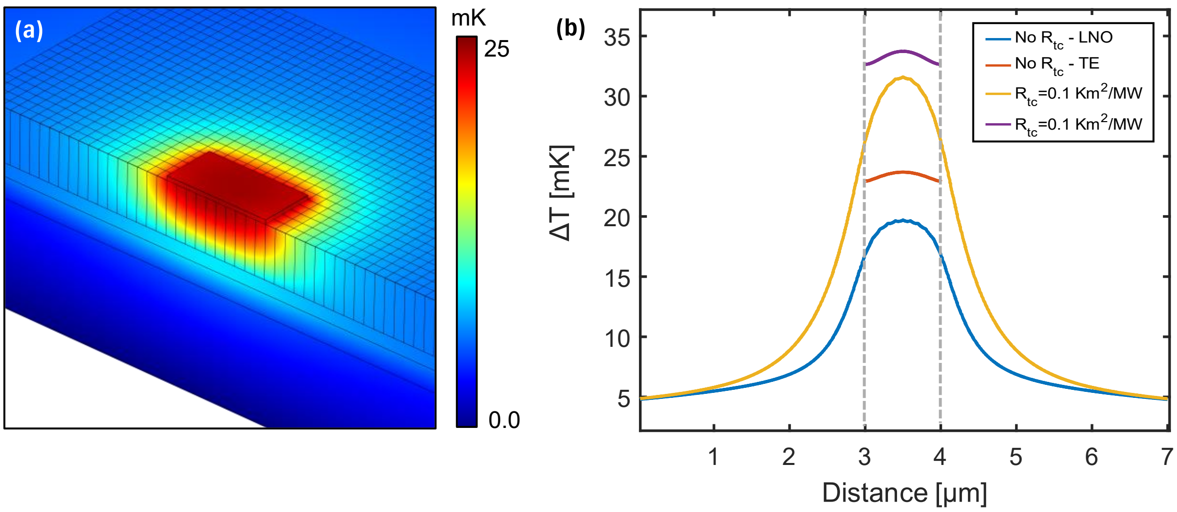

Figure S8 (a) Modelled temperature map for a 1x1 μm^2 lateral dimension device under 5 V bias. The model has been cut cross-sectionally across a plane of symmetry, revealing the interior temperature profile. (b) Line sections of temperature across the top electrode and within the $LiNbO_3$ layer for the cases of no thermal barrier resistances (R_{tc}) considered and for the case of R_{tc} = 0.1 Km^2/MW applied to the top and bottom $LiNbO_3$/electrode interfaces. The vertically running dashed lines indicate the extent of the electrode on the top surface.

Note 3: Modelling of 1x1 μm^2 $LiNbO_3$ domain wall device

We have carried out finite element electrothermal modelling of a $LiNbO_3$ device with a much smaller 1x1 μm^2 Pt electrode with 40 nm thickness, presented in Figure S8. For computational efficiency, the model geometry was cut along a symmetry axis, and both the $LiNbO_3$ substrate and SiO_2 layer thicknesses were reduced by a factor of 10 and their thermal conductivity values increased by the same factor. As before, domain walls were modelled as 2D current carrying planes (using 'electrical shielding' boundary condition), this time arranged in a finer square gridding of 100 nm side (see Figure S8a). To be consistent with the measurements made in the main manuscript, the domain wall electrical conductivity was chosen as σ_0 = 0.35 S/m. For an applied bias condition of 5 V, the current was 118 nA and the dissipated power was 0.59 µW. The effective current density quoted in the main text is obtained by dividing the current by the surface area of the top electrode, giving a value of 118 nA/μm^2. Under these conditions, the peak surface temperature rise is very small, being approximately 20 mK

(Figure S8b). We have also carried out the model with thermal boundary resistance value of 0.1 Km^2/MW attributed to both the top and bottom $LiNbO_3$ interfaces, giving a peak temperature rise of 32 mK (i.e. factor ~1.6 increase), which is still negligible.

Note 4: Calibration of SThM tip temperature

Calibration measurements were carried out by taking temperature readings on the surface of the platinum bar deposited on the lithium niobate sample. This ensures that material-dependent thermal resistance contributions associated with the tip-sample contact will also be captured in the calibration curve. The sample was silver pasted onto the surface of a heated ceramic surface which was temperature controlled by PID feedback using readout from a resistive platinum sensor. The temperature of the stage was increased incrementally, and enough time was allowed for the tip temperature readout to stabilize before it was recorded. For our Asylum MFP-3D system, the SThM mode is proprietary and the user only has access to the software temperature readout panel (not the Wheatstone bridge output voltage). Plotting the known stage temperature (assumed as the sample surface temperature) versus the tip readout temperature gives the plot shown in Figure S8 for two different thermoresistive SThM probes from the same manufacturer. Tip 1 is the probe used for collecting the data in the main manuscript and the gradient of the plot (1.8 ± 0.1) gives the conversion factor for determining sample surface temperature rise from the software readout value.

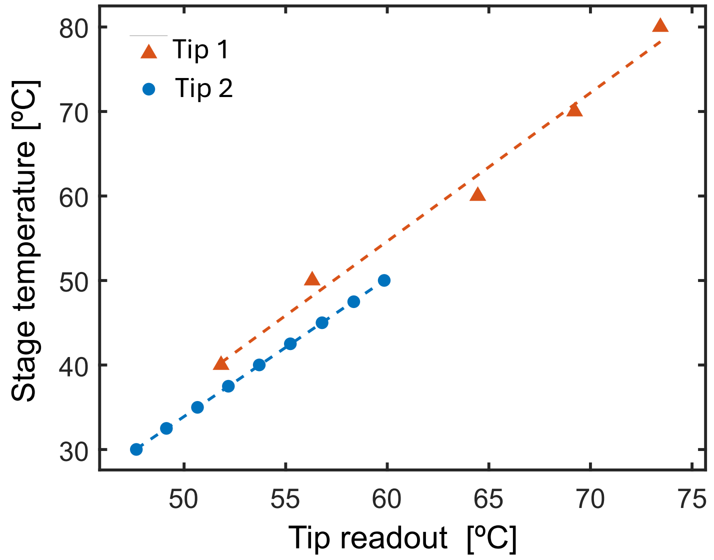

Figure S9 Plot of temperature of the feedback-controlled stage temperature versus the SThM tip readout for two different commercial KNT-SThM-2an SThM tips.

Supporting Information References

(1) N. Harnack, S. Rodehutskors, B. Gotsmann, "Scanning Thermal Microscopy Method for Self-Heating in Nonlinear Devices and Application to Filamentary Resistive Random-Access Memory" *ACS Nano* **2025**, 19, 5342.

(2) F. Menges, P. Mensch, H. Schmid, H. Riel, A. Stemmer, B. Gotsmann, "Temperature mapping of operating nanoscale devices by scanning probe thermometry" *Nat. Commun.* **2016**, 7, 10874.

(3) A. Labuda, R. Proksch, "Quantitative measurements of electromechanical response with a combined optical beam and interferometric atomic force microscope" *Appl. Phys. Lett.* **2015**, 106, 253103.

(4) E. Pop, "Energy dissipation and transport in nanoscale devices" *Nano Res.* **2010**, 3, 147.

(5) S. Deshmukh, M. Muñoz Rojo, E. Yalon, S. Vaziri, C. Koroglu, R. Islam, R. A. Iglesias, K. Saraswat, E. Pop, "Direct measurement of nanoscale filamentary hot spots in resistive memory devices" *Sci. Adv.* **2022**, 8, eabk1514.
